# The Prevalence of Inorganic Mercury in Human Kidneys Suggests a Role for Toxic Metals in Essential Hypertension

**DOI:** 10.3390/toxics9030067

**Published:** 2021-03-21

**Authors:** Roger Pamphlett, Philip A. Doble, David P. Bishop

**Affiliations:** 1 Discipline of Pathology, Brain and Mind Centre, Sydney Medical School, The University of Sydney, Sydney 2050, Australia; 2 Department of Neuropathology, Royal Prince Alfred Hospital, Sydney 2050, Australia; 3 Elemental Bio-Imaging Facility, School of Mathematical and Physical Sciences, University of Technology Sydney, Sydney 2007, Australia; Philip.Doble@uts.edu.au (P.A.D.); David.Bishop@uts.edu.au (D.P.B.)

**Keywords:** mercury, kidney, essential hypertension, environmental toxicity, heavy metal, toxic metal, risk factor, cadmium, elemental analysis, renal cell carcinoma

## Abstract

The kidney plays a dominant role in the pathogenesis of essential hypertension, but the initial pathogenic events in the kidney leading to hypertension are not known. Exposure to mercury has been linked to many diseases including hypertension in epidemiological and experimental studies, so we studied the distribution and prevalence of mercury in the human kidney. Paraffin sections of kidneys were available from 129 people ranging in age from 1 to 104 years who had forensic/coronial autopsies. One individual had injected himself with metallic mercury, the other 128 were from varied clinicopathological backgrounds without known exposure to mercury. Sections were stained for inorganic mercury using autometallography. Laser ablation-inductively coupled plasma-mass spectrometry (LA-ICP-MS) was used on six samples to confirm the presence of autometallography-detected mercury and to look for other toxic metals. In the 128 people without known mercury exposure, mercury was found in: (1) proximal tubules of the cortex and Henle thin loops of the medulla, in 25% of kidneys (and also in the man who injected himself with mercury), (2) proximal tubules only in 16% of kidneys, and (3) Henle thin loops only in 23% of kidneys. The age-related proportion of people who had any mercury in their kidney was 0% at 1–20 years, 66% at 21–40 years, 77% at 41–60 years, 84% at 61–80 years, and 64% at 81–104 years. LA-ICP-MS confirmed the presence of mercury in samples staining with autometallography and showed cadmium, lead, iron, nickel, and silver in some kidneys. In conclusion, mercury is found commonly in the adult human kidney, where it appears to accumulate in proximal tubules and Henle thin loops until an advanced age. Dysfunctions of both these cortical and medullary regions have been implicated in the pathogenesis of essential hypertension, so these findings suggest that further studies of the effects of mercury on blood pressure are warranted.

## 1. Introduction

High systolic blood pressure is the leading risk factor for global disease burden, when ranked by risk-attributable disability-adjusted life-years, and accounts for 10.4 million deaths annually [1]. The cause of most cases of hypertension, however, remains unknown [2]. The kidney has long been considered to play a central role in the pathogenesis of essential hypertension, with the most likely mechanism being impaired renal sodium excretion [3,4]. The initial pathogenetic factors leading to this are unclear, but increased sodium reabsorption in the proximal tubules [5,6], and/or oxidative damage leading to medullary ischemia [7,8], are suspected to play roles in raising blood pressure.

Epidemiological, experimental, and clinical reports suggest an association between hypertension and exposure to mercury [9,10,11,12,13,14,15,16,17,18]. A meta-analysis of 29 studies investigating the relationship between mercury biomarkers and hypertension concluded that mercury is indeed associated with hypertension and that a dose–response relationship exists between the two [9]. Furthermore, hypertension is more common in the high methylmercury exposure area of Minamata in Japan than in a nearby area of low methylmercury exposure [10], and mortality from hypertension is greater in Minamata city than in the surrounding region [11,12]. Several animal studies indicate that exposure to methylmercury gives rise to increased blood pressure [13,14,15], and accidental exposure to mercury in humans can result in increased blood pressure [16]. The mechanisms underlying mercury-induced hypertension remain unclear, but either proximal tubule dysfunction causing sodium retention or the generation of free radicals causing vasoconstriction in the medulla are possibilities [17,18].

Autometallography is a histochemical technique used to locate inorganic mercury within cells [19]. Autometallography-detected mercury has been found in the renal proximal tubules of frogs [20], fish [21], whales [22], mice [23], rats [24,25,26], dogs [27], primates [28], and two humans [29,30] who had been exposed to mercury. Mechanisms and consequences of mercury uptake and elimination in proximal tubules have been studied extensively [31,32,33,34,35,36,37]. Proximal tubules take up mercury at low mercury exposure levels, such as those resulting from the placement of even a few mercury-containing amalgam dental fillings in primates [28] or after exposure to single low doses of inorganic mercury in mice [38]. Rarely have other parts of the kidney, such as the glomeruli, Henle loops, distal tubules, or collecting ducts been shown to contain mercury after experimental exposures, and then only after administration of large doses of mercury [27].

Given the potential roles of both the kidney and of mercury in hypertension, we sought to determine the distribution and the prevalence of mercury in the human kidney. To do this, mercury was located in the kidneys of a large number of people over a wide range of ages, using two elemental bio-imaging techniques, autometallography and laser ablation-inductively coupled plasma-mass spectrometry (LA-ICP-MS).

## 2. Materials and Methods

### 2.1. Ethics

This study (The role of toxic metals in human diseases, X2014-029) was approved by the Human Research Committee, Sydney Local Health District (Royal Prince Alfred Hospital Zone). This institutional review board waived the need for written informed consent from relatives of individuals studied since this was a de-identified retrospective study of archived paraffin-embedded tissue.

### 2.2. Sample Collection

Paraffin-embedded kidney tissue blocks were obtained from the tissue archive of The New South Wales Department of Forensic Medicine. These had been taken as part of standard tissue sampling from the autopsies of 129 people (81 male, 48 female) with a mean age of 54 years, SD 28 years, median 47 years, and age range of 1–104 years. Females had a higher mean age (60 years, SD 31 years) than males (50 years, SD 25 years) (*p* = 0.041). Major pre-mortem medical conditions were: none known (N = 53), neurodegenerative disease (N = 41), psychosis (N = 29), epilepsy (N = 2), and one each of anorexia nervosa, Down syndrome, post-traumatic stress disorder, and self-injection with metallic mercury.

The samples were categorised into two groups: (1) *Known mercury exposure*. A man who injected himself intravenously with metallic mercury and later committed suicide was exposed to a consistently high level of circulating inorganic mercury for 5 months. At autopsy, mercury was found in several of his organs, including the heart and brain [30,39,40,41]. (2) *Unknown mercury exposure*. In 128 people without known sources of mercury exposure, causes of death were: suicide (N = 29), trauma (N = 21), cardiovascular (N = 21), drowning (N = 14), drug overdose (N = 14), infection (N = 10), undetermined (N = 6), choking (N = 4), cerebrovascular (N = 3), respiratory (N = 2), and one each of cancer, asphyxia, sudden unexpected death from epilepsy, and undernutrition.

### 2.3. Autometallography

Paraffin blocks were sectioned at 7 μm with a Feather S35 stainless steel disposable microtome blade, deparaffinised, and stained for inorganic mercury with silver nitrate autometallography, which represents the presence of mercury as black silver grains surrounding the mercury [42]. Autometallography is a sensitive amplification technique that can detect as few as 10 mercury sulphide/selenide molecules in a cell [43]. Sections were placed in physical developer containing 50% gum arabic, citrate buffer, hydroquinone, and silver nitrate at 26 °C for 80 min in the dark; washed in 5% sodium thiosulphate to remove unbound silver; counterstained with mercury-free hematoxylin; and viewed with bright-field microscopy. Each staining run included a control section of mouse spinal cord where motor neuron cell bodies contained mercury following an intraperitoneal injection of mercuric chloride; sections were from archived paraffin blocks of a previously published experiment approved by the Animal Ethics Committee of the University of Sydney [44]. Sections were stained with hematoxylin only to act as a control for the autometallography.

Microscopic identification of different subsets of kidney cells was based on standard histological criteria [45]. To help characterise the cell types in the kidney, autometallography-stained sections from six samples were immunostained with CD10 (Novocastra-Leica, clone 56C6), which stains proximal tubules and collecting ducts prominently [46], using a Leica Bond III staining platform (Leica Biosystems, Melbourne, Australia). Heat-mediated antigen retrieval was undertaken and a 1:25 dilution of the primary antibody was incubated at ambient temperature for 30 min. Bond Polymer Refine Red Detection (DS9390) was used so as not to obscure the black autometallography grains.

### 2.4. Laser Ablation-Inductively Coupled Plasma-Mass Spectrometry (LA-ICP-MS)

To confirm which metal autometallography was demonstrating, since autometallography can also detect inorganic silver and bismuth [47,48], and to look for the presence of other toxic metals, 7 μm paraffin sections of six kidney samples were deparaffinised and subjected to LA-ICP-MS for mercury, silver, bismuth, aluminium, gold, cadmium, chromium, iron, nickel, and lead. Analyses were carried out on a New Wave Research NWR-193 laser or a Teledyne Cetac LSX-213 G2+ laser hyphenated to an Agilent Technologies 7700x ICP-MS, with argon used as the carrier gas. LA-ICP-MS conditions were optimised on NIST 612 Trace Element in Glass CRM and the sample was ablated with a 50 µm spot size and a scan speed of 100 µm/s at a frequency of 20 Hz. The data were collated into a single image file using in-house developed software and visualised using FIJI.

### 2.5. Statistical Analyses

Prism v8.4 software was used for chi-square analyses to compare categorical variables and aging trends, and *t*-tests to compare continuous variables. Significance was assessed at the 0.05 level.

## 3. Results

### 3.1. Distribution of Mercury in the Kidney

#### 3.1.1. Known Mercury Exposure (N = 1)

**Figure 1:**
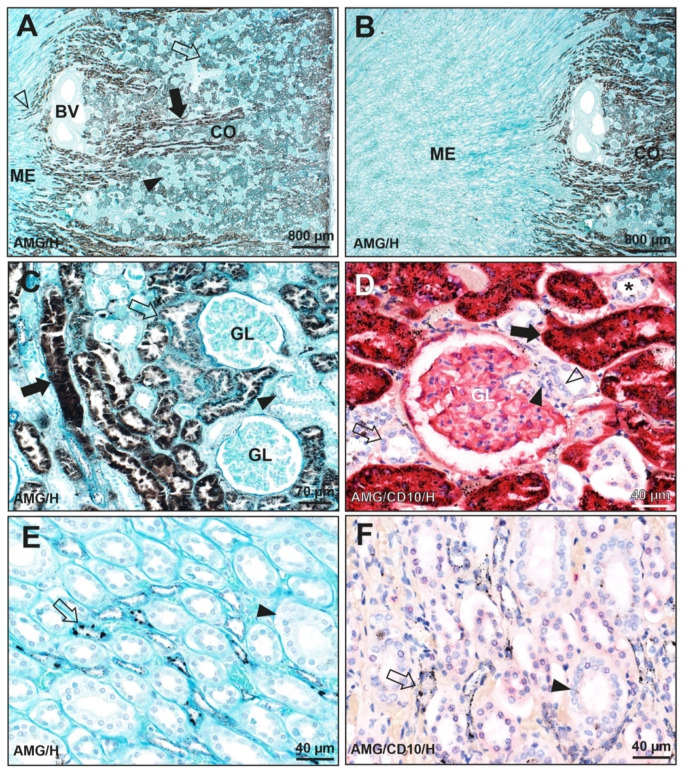
Autometallography of the kidney of a man who had injected himself with metallic mercury (K24). (**A**) Black-staining mercury is seen in the renal cortex (CO) in cells of both the straight (filled arrow) and convoluted (open arrow) proximal tubules, with more mercury in straight tubules. The pale cortical regions (closed arrowhead) contain glomeruli and distal tubules. Some mercury-containing proximal straight tubules (open arrowhead) extend a short distance into the pale-staining medulla (ME). Large pale-staining blood vessels (BV) are present near the cortico-medullary junction. (**B**) A microscopic field to the left of the image in A shows the difference between the dark-staining mercury in the renal cortex (CO) and the pale medulla (ME). (**C**) Mercury is seen in cells of the proximal straight tubules (filled arrow), with less in the proximal convoluted tubules (open arrow). No mercury is seen in two glomeruli (GL) or in distal tubules (arrowhead). (**D**) Red CD10 immunostaining is seen in proximal tubule cells containing black mercury grains (filled arrow). In CD10-negative distal tubules, either no (open arrow) or minimal (asterisk) mercury staining is seen. No mercury is seen in the macula densa (open arrowhead) or Lacis cells (filled arrowhead) of the juxtaglomerular apparatus or in a glomerulus (GL) whose podocytes stain lightly with CD10. (**E**) Cells in Henle thin loops in the medulla contain black mercury granules of varying size (arrow). Collecting tubules (arrowhead) do not contain mercury. (**F**) Cells in Henle thin loops (not CD10-immunostained) in the medulla contain black mercury granules of varying size (arrow). Collecting tubules that stain with CD10 (arrowhead) do not contain mercury. Light brown/yellow staining is from red blood cells in capillaries. AMG/H autometallography/hematoxylin, AMG/CD10/H autometallography/CD10 immunostaining/hematoxylin, K identity number (see Table 1).

In the man who injected himself with metallic mercury, autometallography of the kidney showed black mercury staining dispersed throughout the cytoplasm of proximal tubule cells in the cortex, more in straight than in convoluted tubules (Figure 1). In the medulla, autometallography showed mercury as black particulate deposits in cells of Henle thin loops (Figure 1). No significant mercury was seen in glomeruli, distal tubules, juxtaglomerular apparatus, or collecting ducts.

#### 3.1.2. Unknown Mercury Exposure (N = 128)

**Figure 2:**
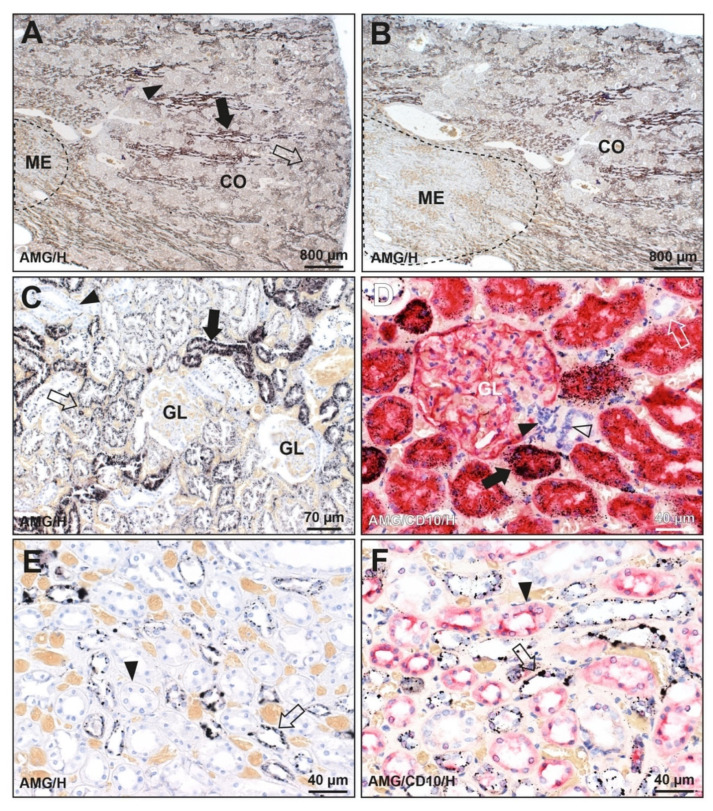
Mercury in the kidney of a man with no known mercury exposure (K44). (**A**) Black-staining mercury is seen in the renal cortex (CO) in cells of the straight (filled arrow) and convoluted (open arrow) proximal tubules, with more mercury in straight tubules. The pale cortical regions (arrowhead) contain glomeruli and distal tubules. The medulla (ME, dashed outline) shows no mercury staining at this magnification. (**B**) A microscopic field to the left of the image in A shows the difference between the plentiful mercury in the renal cortex (CO) and no obvious mercury staining in the medulla (ME, dashed outline). (**C**) Mercury is seen in cells of the proximal straight tubules (filled arrow), with less in the proximal convoluted tubules (open arrow). No mercury is seen in two glomeruli (GL), or in distal tubules (arrowhead). (**D**) Red CD10 immunostaining shows proximal tubule cells containing black mercury grains (filled arrow). No mercury is seen in CD10-negative distal tubules (open arrow), in the macula densa (open arrowhead) or Lacis cells (filled arrowhead) of the juxtaglomerular apparatus or in a glomerulus (GL) whose podocytes stain lightly with CD10. (**E**) Cells in Henle thin loops in the medulla contain black mercury granules of varying size (arrow). Collecting tubules (arrowhead) do not contain mercury. (**F**) Cells in Henle thin loops (not CD10-immunostained) in the medulla contain mercury granules of varying size (arrow). Collecting tubules that stain with CD10 (arrowhead) do not contain mercury. Light brown/yellow staining is from red blood cells in capillaries. AMG/H autometallography/hematoxylin, AMG/CD10/H autometallography/CD10 immunostaining/hematoxylin. K identity number (see Table 1).

**Figure 3:**
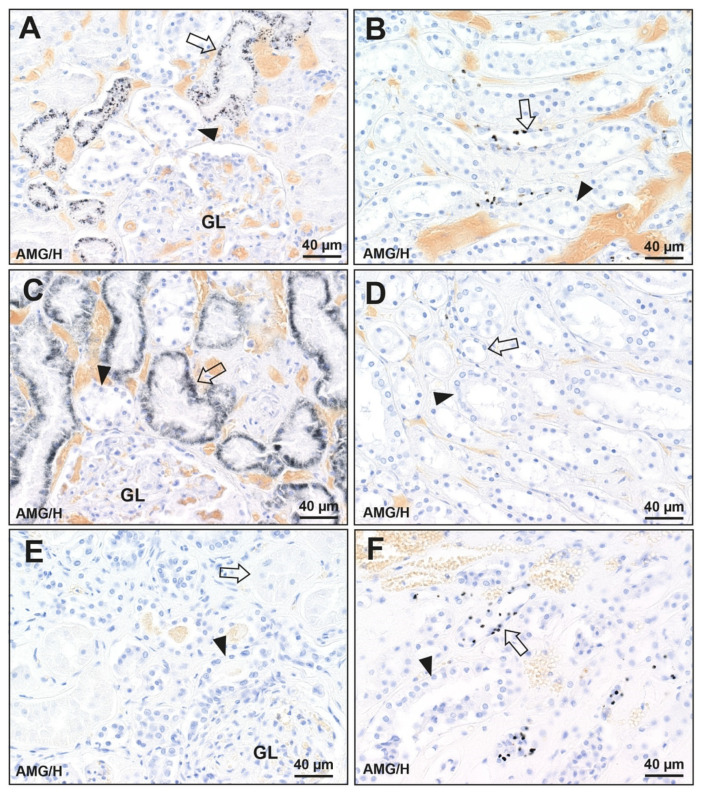
Patterns of mercury distribution in three kidneys (no known mercury exposure). (**A**,**B**) Mercury in the cortex and medulla (K19). (**A**) In the cortex, black-staining mercury is seen in cells of proximal tubules (arrow) but not distal tubules (arrowhead) or glomeruli (GL). (**B**) In the medulla, discrete mercury granules are seen in cells of Henle thin loops (arrow) but not in collecting ducts (arrowhead). (**C**,**D**) Mercury in the cortex only (K79). (**C**) In the cortex, mercury is seen in cells of proximal tubules (arrow) but not distal tubules (arrowhead) or glomeruli (GL). (**D**) In the medulla, no mercury is seen in cells of Henle thin loops (arrow) or collecting ducts (arrowhead). (**E**,**F**) Mercury in the medulla only (K101). (**E**) In the cortex, no mercury is seen in cells of proximal tubules (arrow), distal tubules (arrowhead), or glomeruli (GL). (**F**) In the medulla, mercury is seen in cells of Henle thin loops (arrow) but not in collecting ducts (arrowhead). AMG/H autometallography/hematoxylin, K identify number (see Table 1).

Three patterns of mercury staining were found in the kidneys of the 128 people without known mercury exposure (Figure 2 and Figure 3 and Table 1). (1) Mercury was seen in cells of proximal tubules of the cortex as well as in Henle thin loops of the medulla in 32 of the 128 (25%) kidneys, with more mercury in the proximal convoluted than straight tubules. This was the same pattern seen in the man who injected himself with mercury. (2) Mercury was present in proximal tubules only in 21 of the 128 (16%) kidneys. (3) Mercury was seen in Henle thin loops only in 29 of the 128 (23%) kidneys. Mercury was not seen in glomeruli, distal tubules, juxtaglomerular apparatus, or collecting ducts.

### 3.2. Prevalence of Mercury in the Kidney

**Figure 4:**
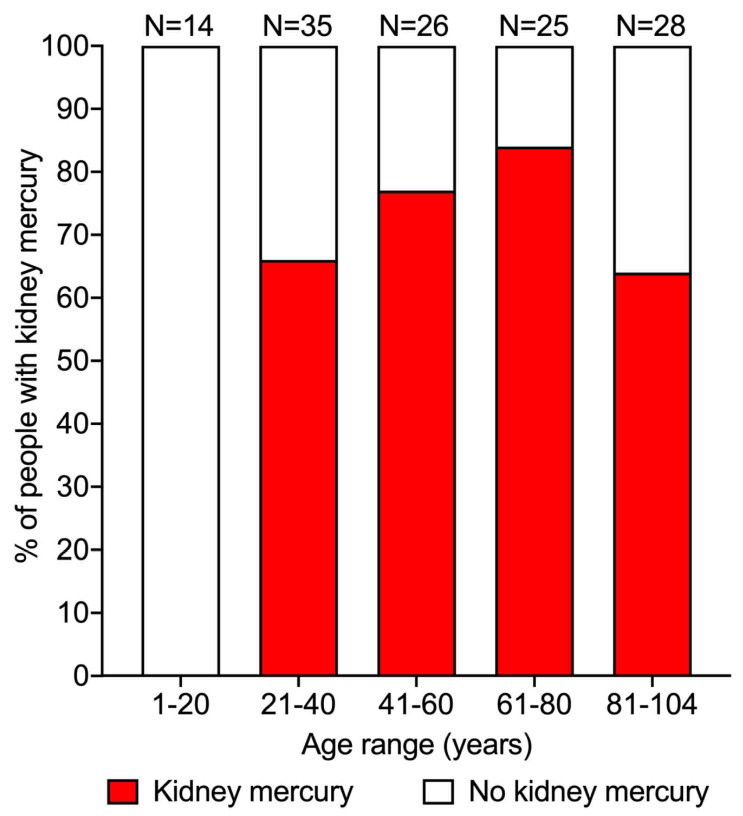
Prevalence of mercury in the human kidney at different ages. No kidney mercury was seen in the first two decades of life. In the 41–60 years age range, mercury was found in 66% of people, rising to 77% in the 41–60 years group and 84% in the 61–80 years group, before falling to 64% in the 81–104 years age range. Numbers above bars = numbers in age groups.

Overall, mercury (either cortical or medullary) was detected on autometallography in the kidneys of 82 of the 128 people (64%) without known mercury exposure. The proportion of people who had mercury in their kidneys varied in different age ranges (Figure 4). People in the first two decades of life had no kidney mercury, followed by 66% of people with kidney mercury in the subsequent 21–40 years age group. The prevalence of kidney mercury increased to 77% in the 41–60 years group, reaching a maximum of 84% of people in the 61–80 years group, then falling back to 64% of people in the final 81–104 years group. The overall trend for aging to increase the proportion of mercury-positive kidneys was significant (*p* < 0.0001).

The 48 females in the study had a slightly (non-significant) higher proportion of mercury-positive kidneys (69%) compared to the 80 males (61%), probably because females had a higher mean age (60 years SD 31 years) than males (50 years SD 25).

### 3.3. Metals Detected in the Kidney on LA-ICP-MS

**Figure 5:**
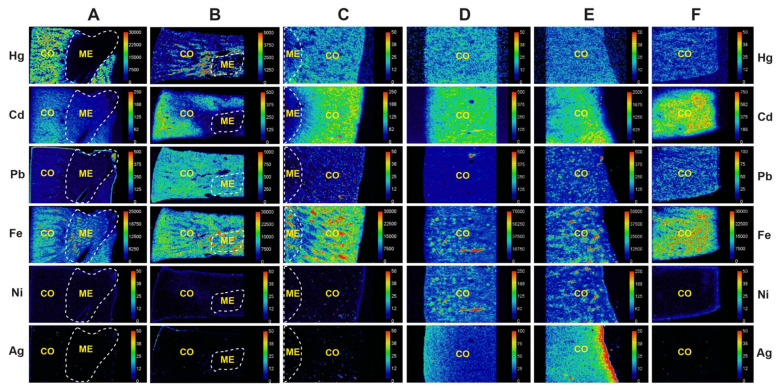
LA-ICP-MS detection of metals in six kidney samples. (**A**) AMG+ve cortex and medulla (K16). Mercury is detected in the cortex but not the medulla. Cadmium is present in the cortex, and iron in the cortex and medulla. (**B**) AMG+ve cortex and medulla (K32). Mercury is seen in the cortex, but not in the medulla. Cadmium, lead, and iron are seen in the cortex, and lead and iron in the medulla. (**C**) AMG+ve cortex and medulla (K19). Mercury, cadmium, and iron are seen in the cortex, and iron in the medulla. (**D**) AMG+ cortex (K69). Mercury, cadmium, iron, nickel, and silver are present in the cortex. (**E**) AMG-ve cortex (K51). Mercury, cadmium, lead, iron, nickel, and silver (with edge effect) are seen in the cortex. (**F**) AMG-ve cortex (K39). Mercury, cadmium, lead, and iron are present in the cortex. Scale = counts per second (proportional to abundance). CO cortex, ME medulla (within dashed outlines), AMG autometallography, K identify number (see Table 1).

LA-ICP-MS of six kidney samples (three with cortex only in the field of view) confirmed the presence of mercury in the cortex of four samples that stained positively for inorganic mercury with autometallography (Figure 5, Table 2). In two samples where autometallography did not detect mercury in the cortex, mercury was seen on LA-ICP-MS, indicating the presence of organic mercury, which is not detected by autometallography. The cortex of all six samples contained cadmium and two contained silver. Lead was seen in the cortex of three samples and in the medulla of one. Iron was widespread in the cortex and medulla, probably due to iron in intravascular red blood cells. The distribution of nickel in the cortex of two samples was similar to that of iron, suggesting this too was due to circulating metal. Despite mercury being found on autometallography in scattered Henle thin loops, no mercury was detectable in the medulla on LA-ICP-MS, probably because of the higher sensitivity of mercury detection by autometallography compared to LA-ICP-MS [49]. No chromium, aluminium, bismuth, or gold was seen in any kidneys (data not shown).

## 4. Discussion

Key findings of this study are that mercury was found commonly in the proximal tubules and Henle thin loops of human adult kidneys, and that the proportion of people with mercury in their kidneys increased throughout most of adult life. In addition, several other toxic metals, most commonly cadmium and lead, were found in some kidneys.

**Figure 6:**
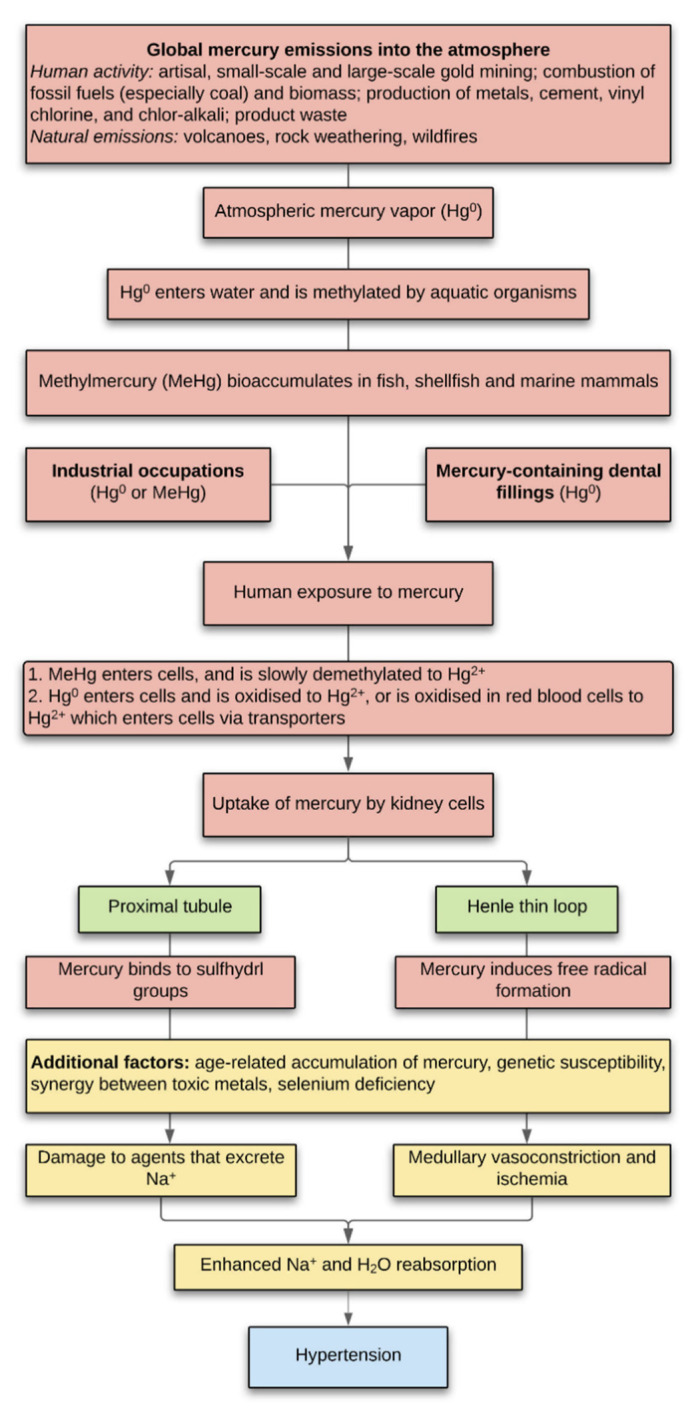
Potential mechanisms of kidney mercury-induced hypertension. Exposure to mercury results in organic and inorganic mercury being taken up by the cells of proximal tubules and/or Henle thin loops. In proximal tubules of the cortex, mercury-initiated damage to agents that excrete sodium in response to elevated blood pressure (for example by selective binding of mercury to sulfhydryl-rich proteins) would enhance sodium reabsorption. In the medulla, free radicals induced by mercury in Henle thin loops could result in medullary ischemia, also with enhancement of sodium reabsorption. Both or either of these cortical and medullary mechanisms would increase sodium and concomitant water reabsorption, with resultant hypertension. Mercury toxicity would be accentuated by bioaccumulation of mercury over time, genetic susceptibilities to mercury toxicity, the presence of other heavy metals, or deficiencies in mercury-protective mechanisms such as selenium.

All humans are exposed to mercury emitted into the environment from both anthropogenic and natural sources (Figure 6) [50]. Common human exposures to mercury are from consuming mercury-contaminated fish, occupations such as gold mining, and from mercury-containing dental amalgam fillings [51]. Methylmercury crosses cell membranes readily, mostly by the formation of methylmercury-cysteine complexes that enter cells on neutral amino acid carriers [52] and is slowly converted in cells into more toxic inorganic mercury (Hg^2+^) [53]. Mercury vapor also passes through the cell membrane freely and is oxidised to Hg^2+^ within the cell or is oxidised in circulating red blood cells to Hg^2+^, which crosses some cell membranes (such as those of renal tubules) via transporters [52,54]. Once inside the cell, mercury attaches preferentially to intracellular membranous structures such as lysosomes, mitochondria, and the nuclear envelope [55].

Chemical analyses of mercury in human kidneys report more mercury in people who had mercury amalgam dental fillings [56,57,58]. Autometallography of kidney tissue sections from single individuals found mercury in renal tubules both 5 months [30] and 17 years [29] after mercury exposure. The renal cortex appeared to contain more mercury on atomic absorption in eight people who committed suicide than in 10 others [59], though case numbers were small and the authors could not rule out the role of chance. In our study, there was no significant difference in the proportion of people with mercury in their kidneys who committed suicide (N = 19 of 29, 66%) compared to others (N = 64 of 100, 64%).

The location of mercury in renal proximal tubules and Henle thin loops suggests two pathways by which mercury in the kidney could contribute to essential hypertension (Figure 6): (1) Several studies have stressed the importance of the role proximal tubules play in the pathogenesis of hypertension [5,6,60,61]. Mercury could preferentially damage humoral or hormonal agents that decrease ion transport in the proximal tubule [62], with resultant increased reabsorption of sodium and water. One mechanism could be that mercury, which has an affinity for sulfhydryl groups (found mostly in cysteine) can selectively inactivate proteins with a high sulfhydryl content [63]. A comparison of the sulfhydryl content of the humoral and hormonal agents that either decrease or increase ion transport in the proximal tubule [62] could provide evidence to support this hypothesis. (2) Experimental evidence in rats indicates that oxidative stress in the renal medulla results in vasoconstriction and medullary ischemia, which leads to enhanced sodium and water reabsorption and subsequent hypertension [8,64]. In these rats, it is suggested that reactive oxygen species are released by the Henle thick ascending limbs into surrounding capillaries. In our human samples, mercury was located in the Henle thin loops, and since mercury is known to promote oxidative stress [37,65,66], a similar mechanism of medullary ischemia could lead to human hypertension. Both these damaging effects of mercury within the kidney would be augmented by bioaccumulation of the metal with aging [67], genetic susceptibilities to mercury toxicity [68], the presence of other heavy metals [69,70], and a lack of mercury-protective selenium [71].

The finding that mercury is found commonly in adult human kidneys could help explain several epidemiological findings in hypertension [72,73,74,75,76,77,78,79,80,81]. (1) The incidence of hypertension rises with age [72,82], so it was of interest that in our study, the proportion of people with mercury in their kidneys also increased with age, at roughly the same rate. In our final age group of people over the age of 80 years, the proportion with kidney mercury fell back, which suggests a “survivor” effect, possibly because people who have been exposed to less mercury during their lives would tend to live longer [83]. (2) Younger men have higher blood pressure than younger women on average, and in some animals, males are predisposed to higher blood pressures than females [73,74]. One factor that could contribute to this gender difference is that the kidney of the male mouse takes up more of a given dose of mercury than does the female kidney [84]. Unfortunately, we did not have quantitative data from our project that could assess whether more mercury was present in male than female kidneys. (3) Renal cell carcinoma arises from proximal tubule cells, and appears to be associated with hypertension [75,76]. It may therefore be relevant to the pathogenesis of renal cell carcinoma that our study showed that human proximal tubules commonly contain mercury, which is genotoxic [85,86]. In addition to causing somatic mutations in adult kidney cells, mercury in proximal tubule progenitor cells in the foetus could be genotoxic since mercury in non-toxic doses passes through the placenta and enters foetal renal tubules [87]. It would be of interest to assess how often proximal tubules adjacent to human renal cell carcinomas contain mercury, in the same way as has been done in breast and pancreatic cancers [49,88]. However, because mercury is so commonly found in adult human proximal tubules, large numbers of tumour and non-tumour samples would be needed to assess whether kidney mercury is in fact associated with renal cell carcinoma. (4) Firefighters who worked for 10 years or more with wildfires have greater odds of being diagnosed with hypertension than those working fewer than 10 years with wildfires [77]. So it is worth noting that wildfires have long been recognised as a source of mercury emissions [89,90], especially if they affect regions where the soil has previously been polluted with mercury from activities such as gold mining [91]. (5) People who live in the vicinity of volcanoes tend to have higher blood pressures [78,79], and volcanic eruptions are sources of mercury [92]. (6) Exposure to severe particulate atmospheric pollution has been linked to higher blood pressure [80,81], and atmospheric pollution often contains mercury [93].

Estimates of mean blood pressure by world region indicate that the prevalence of high blood pressure has decreased over time in regions such as North America and Western Europe, but has increased over time in others such as China, India, and Southeast Asia [94]. Several factors could underlie this regional heterogeneity in hypertension prevalence, such as variations in sodium uptake [94]. Of note, however, geographic regions of increases and decreases in hypertension prevalence over time [94] overlap with regions where increases and decreases of anthropogenic emissions of mercury have been reported [50], as well as with regions with increases and decreases in discharges of mercury into rivers [95]. For example, the United States has had reductions in mercury atmospheric emissions and discharges of mercury into rivers, and a decreased prevalence of hypertension; on the other hand, China and India have had increases in mercury atmospheric emissions and discharges of mercury into rivers, and an increased prevalence of hypertension [50,94,95]. Mercury exposure, therefore, needs to be considered when assessing possible reasons for the variation in the prevalence of hypertension between different world regions.

Increased retention of sodium and water due to renal dysfunction is not the sole mechanism suspected to underlie essential hypertension, since noradrenaline excess causing increased sympathetic output is a frequent finding in people with raised blood pressure [96,97,98]. This is reflected in medication regimens used to treat hypertension, which often include diuretics to promote natriuresis combined with beta-adrenoreceptor blockers that reduce sympathetic overactivity [99]. Recent work has shown that mercury is found commonly in the adult human adrenal medulla and could lead to increased noradrenaline output [100]. This raises the possibility that two hits of mercury, one in the kidney and one in the adrenal medulla, could underlie the combined renal and sympathetic dysfunction found in many people with essential hypertension.

Toxic metals other than mercury that were found in our kidney samples were cadmium, lead, and silver. Several epidemiological studies have examined possible links between serum or urine cadmium levels and hypertension, but with inconsistent results, leading to calls for future longitudinal studies [18,101]. Increased levels of lead in blood [102,103,104] and bone [105] have been associated with hypertension. Acute exposure to silver nitrate causes a decrease in blood pressure, and silver is not currently thought to be toxic to the cardiovascular system [106]. Synergistic toxic effects have been described for a range of heavy metals, especially mercury and cadmium [70,107]. It is, therefore, of interest that some of our kidney samples had mercury, cadmium, and lead together in the cortex. The effects of these metal interactions on the kidney are complex, however, at least in the rat, where paradoxical decreases of hypertensive effects have been described for mercury/lead combinations [69]. We were unable to determine the physiological role of mercury and these other metals on blood pressure in this autopsy study, and further experiments will be required to measure the effects on blood pressure of long-term exposure to single and combinations of toxic metals.

It has long been known that selenium interacts with mercury and appears to decrease mercury toxicity [108,109,110,111,112,113]. More recently, it has been proposed that one deleterious effect of mercury could be its binding to selenium, thus reducing the ability of selenium to participate in selenoenzyme activity [114,115,116]. The kidneys of most people appear to have enough selenium to detoxify mercury [117,118,119], though the trapping of the freely available renal selenium by mercury may have adverse effects [119]. These kidney studies have relied on chemical analyses, so the mercury and selenium levels in individual cells could not be measured. One way of assessing the mercury–selenium status of individual cells is by synchrotron X-ray fluorescence microscopy, which has detected the equivalent of 1:1 mercury–selenium molar ratios within individual neurons [120]. However, this technique requires frozen sections and allows only a small window of tissue sampling. We were unable to reliably measure selenium in the current project, since trace selenium analysis is often refractory to LA-ICP-MS imaging using standard single-quadrupole MS technology, due to polyatomic interferences from the large volumes of argon gas used in creating the plasma and as the carrier gas. Future studies using triple quadrupole-ICP-MS would be needed for the accurate determination of renal selenium levels [121]. A complicating factor in unravelling the relationship between selenium and mercury toxicity is that genetic polymorphism may affect selenium status and responses to selenium therapy [122], so future studies in this field may need to take these genetic variants into account. Studies of the relationship between human selenium levels and hypertension have given mixed results. A recent study of Inuit in Canada suggested that high selenium exposure decreased the risk of hypertension [123], but high serum selenium levels have been associated with an increased prevalence of hypertension [124,125], and most workers are of the opinion that further studies of the effects of selenium on hypertension are needed [126].

This study has several limitations. (1) This was a retrospective forensic/coronial autopsy study, so we did not have access to detailed clinical medical information to allow us to determine whether individuals had been diagnosed with hypertension during life or if they had been taking antihypertensive medication. Large prospective autopsy studies of people with known blood pressure recordings would be needed to ascertain a link between kidney mercury and hypertension. In such a study, further information gathered could include renal function tests, blood, urine, hair and toenail levels of toxic metals, selenium levels, and whole-genome analyses to look for genetic susceptibility variants. (2) We did not have access to occupational data, places lived, dental records, or dietary habits to assess whether individuals had any known sources of mercury exposure. However, we do know from a previous study that over 90% of Australians over the age of 40 years eat seafood regularly and that over 80% have mercury-containing dental fillings [127], both common sources of human exposure to mercury [51]. (3) We had only modest numbers of individuals in the 0–20 years group. Hypertension is unusual at this age [82], and large numbers of samples would be needed to give an accurate estimation of the proportion of people in this early age group who have kidney mercury. (4) Forensic/coronial autopsy populations, aimed largely at investigating unnatural deaths, cannot exactly replicate conditions in general populations. We tried to minimise the differences by studying people with a range of disorders, as well as those without known medical conditions who died suddenly and unexpectedly. (**5**) We were unable to quantify the amount of mercury in the kidney using these techniques so the results are qualitative in nature.

## 5. Conclusions

In conclusion, mercury is found commonly in the proximal tubules and Henle thin loops of adult human kidneys and increases in aging kidneys until an advanced age. Dysfunctions of both these kidney regions have been implicated in the pathogenesis of essential hypertension. Our study was on human autopsy tissue, so the functional implications of our findings will require confirmation with future experimental studies of the effects of renal toxic metals on blood pressure. Precautionary measures to lessen the possibility of mercury-induced hypertension would include making efforts to reduce the burning of fossil fuels such as coal, reduce artisanal gold mining, limit the consumption of fish, such as shark and swordfish, that contain more mercury than selenium [128], consider alternatives to mercury-containing amalgam dental fillings and ensure an adequate intake of selenium-containing foods [129].

## Figures and Tables

**Table 1:** Mercury (autometallography (AMG) staining) in the proximal tubules and Henle thin loops of 129 kidneys.

ID No.	Age	Sex	Proximal Tubule AMG	Henle Loop AMG	ID No.	Age	Sex	Proximal Tubule AMG	Henle Loop AMG	ID No.	Age	Sex	Proximal Tubule AMG	Henle Loop AMG
K1	1	F	−	−	K44	39	M	+	+	K87	69	M	−	−
K2	2	M	−	−	K45	39	M	−	+	K88	70	M	−	−
K3	2	F	−	−	K46	39	M	−	−	K89	70	M	+	+
K4	2	F	−	−	K47	39	M	+	+	K90	71	F	−	+
K5	3	M	−	−	K48	40	F	−	+	K91	72	F	+	+
K6	4	M	−	−	K49	40	F	+	+	K92	72	F	+	+
K7	9	M	−	−	K50	40	M	−	+	K93	74	F	−	+
K8	16	M	−	−	K51	41	M	−	−	K94	75	M	−	−
K9	18	M	−	−	K52	41	F	−	−	K95	76	F	+	+
K10	18	F	−	−	K53	41	M	−	+	K96	76	F	+	+
K11	18	F	−	−	K54	42	M	+	+	K97	77	F	+	−
K12	20	M	−	−	K55	43	M	−	−	K98	77	M	+	−
K13	20	F	−	−	K56	43	M	−	−	K99	79	M	−	+
K14	20	M	−	−	K57	44	M	−	+	K100	80	F	+	+
K15	23	M	−	−	K58	44	M	+	−	K101	80	M	−	+
K16	24	M *	+	+	K59	45	M	−	+	K102	81	M	+	+
K17	24	M	−	+	K60	45	M	+	−	K103	83	M	+	+
K18	25	F	−	−	K61	45	M	−	−	K104	85	M	−	−
K19	25	M	+	+	K62	46	M	+	−	K105	86	M	+	+
K20	26	F	+	+	K63	46	F	+	+	K106	86	F	+	+
K21	28	M	−	−	K64	46	M	+	−	K107	87	M	−	−
K22	29	F	−	−	K65	47	M	−	+	K108	87	F	−	+
K23	29	M	+	−	K66	47	M	+	+	K109	89	F	−	+
K24	30	M	−	−	K67	48	F	+	+	K110	95	F	−	−
K25	30	M	−	+	K68	49	F	+	+	K111	95	F	−	+
K26	30	M	−	−	K69	49	M	+	+	K112	95	F	−	+
K27	31	M	+	−	K70	49	M	+	−	K113	95	M	−	−
K28	32	M	−	+	K71	49	M	+	+	K114	95	M	−	+
K29	32	M	+	−	K72	53	M	−	+	K115	95	F	+	−
K30	33	F	−	−	K73	55	M	−	+	K116	95	M	−	−
K31	33	M	−	−	K74	58	M	−	−	K117	96	F	−	−
K32	34	M	−	−	K75	59	F	+	+	K118	96	M	+	+
K33	35	M	+	+	K76	59	M	+	+	K119	96	M	+	−
K34	35	F	+	−	K77	61	M	+	−	K120	96	F	+	−
K35	35	F	−	−	K78	61	M	+	+	K121	96	F	−	−
K36	36	F	+	+	K79	61	M	+	−	K122	97	F	−	+
K37	36	M	−	+	K80	61	M	−	−	K123	97	F	+	+
K38	37	M	+	−	K81	61	F	+	+	K124	98	M	−	−
K39	38	M	−	−	K82	62	M	−	+	K125	98	M	−	+
K40	38	F	+	+	K83	63	F	−	+	K126	99	F	+	−
K41	38	M	+	−	K84	66	M	+	−	K127	100	M	−	−
K42	38	M	+	−	K85	67	F	+	+	K128	104	F	−	−
K43	38	F	−	+	K86	67	M	−	+	K129	104	F	−	+

AMG autometallography, ID no. identity number, F female, M male, * mercury self-injection (K16).

**Table 2:** Metals detected by laser ablation-inductively coupled plasma-mass spectrometry (LA-ICP-MS) in six human kidneys.

ID	Site	AMG	LA-ICP-MS									
			Hg	Cd	Pb	Fe	Ni	Ag	Cr	Al	Bi	Au
K16	Cortex	Positive	+	+	−	+	−	−	−	−	−	−
	Medulla	Positive	−	−	−	+	−	−	−	−	−	−
K32	Cortex	Positive	+	+	+	+	+	−	−	−	−	−
	Medulla	Positive	+	−	+	+	+	−	−	−	−	−
K19	Cortex	Positive	+	+	−	+	−	−	−	−	−	−
	Medulla	Positive	−	−	−	+	−	−	−	−	−	−
K69	Cortex	Positive	+	+	+	+	−	+	−	−	−	−
K51	Cortex	Negative	+	+	+	+	+	+	−	−	−	−
K29	Cortex	Negative	+	+	+	+	−	−	−	−	−	−

AMG autometallography, ID identity number, + detected, − not detected, K identity number (see Table 1).
